# Extended Germline Profiling of Cancer Predisposition and Homologous Recombination Repair Genes in 275 Russian Patients with Triple-Negative Breast Cancer

**DOI:** 10.3390/biomedicines14081742

**Published:** 2026-08-01

**Authors:** Peter Alekseevich Shatalov, Anna Aleksandrovna Bukaeva, Egor Mikhailovich Veselovsky, Alexey Aleksandrovich Traspov, Maria Alexandrovna Revkova, Polina Vladimirovna Mishakova, Elena Alekseevna Kirillova, Maria Pavlovna Raygorodskaya, Daria Valerievna Bagdasarova, Irina Alexeevna Leukhina, Anna Petrovna Shinkarkina, Alena Valerevna Murzaeva, Yulia Arturovna Mechenici, Andrei Dmitrievich Kaprin, Petr Viktorovich Shegai

**Affiliations:** 1 National Medical Research Radiological Centre of the Ministry of Health of the Russian Federation, Koroleva st. 4, 249036 Obninsk, Russia; 2 Scientific and Educational Resource Center for Innovative Technologies of Immunophenotyping, Digital Spatial Profiling and Ultrastructural Analysis, Patrice Lumumba Peoples’ Friendship University of Russia (RUDN University), 117198 Moscow, Russia

**Keywords:** triple-negative breast cancer, germline mutations, *BRCA1*, *BRCA2*, homologous recombination, PARP inhibitors, next-generation sequencing

## Abstract

**Background/Objectives:** Triple-negative breast cancer (TNBC) is enriched for germline pathogenic variants in *BRCA1/2* and other DNA repair genes, but the additional value of extended germline profiling beyond *BRCA1/2* remains insufficiently characterized in Russian patients. We aimed to characterize germline pathogenic/likely pathogenic variants (GPV/LPVs) and variants of uncertain significance (VUSes) in cancer predisposition and homologous recombination repair (HRR)-related genes in Russian TNBC patients and to assess the additional yield of extended germline profiling beyond *BRCA1/2*. **Methods:** We retrospectively analyzed germline DNA from 275 patients with histologically confirmed TNBC. Exome sequencing was performed for 204 patients and focused HRR-panel testing for 71 patients on the MGISEQ-G400 platform. **Results:** Overall, 114 patients (41.5%) harbored at least one germline GPV/LPV. *BRCA1/2* GPV/LPVs were detected in 53 patients (19.3%), with *BRCA1* predominating over *BRCA2* (48 vs. 5 carriers) and the recurrent *BRCA1* c.5329dup variant accounting for 23 cases. In the exome subset, 79 of 204 patients (38.7%) carried a GPV/LPV in Groups 1–3, including 48 (23.5%) with BC-related GPV/LPVs; non-*BRCA* Group 1 genes contributed seven additional carriers beyond *BRCA1/2*-only analysis. The expanded HRR set identified 52 GPV/LPV carriers (25.5%) versus 45 (22.1%) in the reference HRR panel, while the common HRR gene set identified 68 carriers (24.7%) in the full cohort. In addition, 183 unique VUSes were found in Groups 1–3 in the exome subset, affecting 128 patients (62.7%). **Conclusions:** Russian TNBC patients show a substantial germline GPV/LPV burden dominated by *BRCA1/2* alterations and the recurrent *BRCA1* c.5329dup founder variant. Extended germline profiling identified additional non-*BRCA* and HRR-related findings beyond *BRCA1/2*, but the incremental yield was moderate and accompanied by a considerable VUS burden. Broader germline testing may therefore support hereditary risk assessment and exploratory HRR-focused stratification, but non-*BRCA* HRR findings should not be considered sufficient for therapy selection without additional tumor-level or clinical evidence.

## 1. Introduction

Breast cancer (BC) is the most frequent malignancy in women and the leading cause of female cancer mortality [1]. Up to 15% of patients with BC have a hereditary predisposition [2], mostly due to germline pathogenic variants (GPVs) in homologous recombination repair (HRR)-associated genes, of which *BRCA1* and *BRCA2* are the most renowned and highly penetrant [3]. Triple-negative BC (TNBC), characterized by the absence of estrogen receptor (ER), progesterone receptor (PR), and human epidermal growth factor receptor 2 (HER2), is the most formidable BC subtype, with high recurrence and mortality rates and limited treatment options [4,5]. In recent years, poly (ADP-ribose) polymerase (PARP) inhibitors (PARPis) have emerged as an effective alternative to cytotoxic chemotherapy of TNBC [5,6,7].

The mechanism of anti-cancer therapy with PARPis is based on synthetic lethality caused by inhibition of PARP-mediated DNA repair. In tumors exhibiting homologous recombination deficiency (HRD), including HRD caused by GPVs affecting the HRR system, PARP inhibition leads to accumulation of DNA damage that cannot be efficiently repaired, resulting in cancer cell death. Several PARPis, including olaparib and talazoparib, have been approved by the FDA for clinical application in BC patients with GPVs in *BRCA1/2* [8,9]. Moreover, accumulating evidence suggests that alterations in other HRR-related genes may also contribute to PARPi sensitivity in selected tumor types; for example, the FDA has approved olaparib for metastatic prostate cancer patients harboring alterations in a broader set of HRR genes [10]. However, in BC patients, the spectrum of HRD-causing GPVs in HRR-related genes beyond *BRCA1/2* and their impact on PARPi efficacy have still not been fully studied, and there is no professional consensus on which genes to investigate [11,12]. According to the recent data across populations, up to ⅓ of GPVs found in the BC patients are located in HRR-associated genes other than *BRCA1/2*, with predominating findings in *ATM*, *CHEK2*, *PALB2*, *RAD51B/C/D*, etc. [13,14,15]. Yet germline genetic testing in a clinical setting is usually restricted to *BRCA1/2* plus several other well-established genes, such as *PALB2*, *CHEK2*, and *ATM*, and in case of TNBC, given its high *BRCA1/2* mutational rate compared to other BC subtypes (14.6% vs. 5%, respectively, without selection for family history [16,17]), genetic testing may often be restricted to *BRCA1/2* only [12,18]. Meanwhile, extended genetic profiling with a focus on GPVs in HRR-associated genes is important for gathering evidence and integrating the scattered data on genotype–phenotype correlations, which modulate not only PARPi efficacy, but also hereditary risks [19,20]. At the same time, extensive genetic tests have become easily conducted as the availability of massive parallel sequencing becomes ubiquitous [21]. In Russia and other Central/Eastern European populations, germline *BRCA1/2* testing in breast cancer has historically been strongly influenced by recurrent founder variants, particularly *BRCA1* c.5329dup/c.5266dupC (5382insC) [22,23]. While *BRCA1/2* founder variants and mixed breast cancer cohorts, including broader DNA repair panels, have been studied in Russian patients [24,25], TNBC-specific data on the germline HRR spectrum beyond *BRCA1/2* remain limited. In the present study, we retrospectively analyzed germline sequencing data from 275 patients with TNBC who underwent genetic testing, investigating the landscape of GPV/LPVs and potentially protein-altering VUSes in cancer predisposition and HRR-related genes. We aimed to evaluate the additional yield of extended germline profiling beyond *BRCA1/2* testing and to explore how often germline HRR-related findings beyond *BRCA1/2* could provide information relevant to research-oriented HRR/PARPi-related therapeutic interpretation.

## 2. Materials and Methods

This retrospective study included 275 patients with histologically confirmed triple-negative breast cancer (TNBC) who underwent germline genetic testing using peripheral blood samples at the National Medical Research Radiological Centre between 2021 and 2025. Patients were identified from routine molecular diagnostic testing performed in the Centre’s molecular diagnostics unit, which receives samples from multiple clinical departments and referring medical institutions. Accordingly, the cohort represents a retrospective diagnostic/referral-center series of TNBC patients with available germline sequencing data rather than a population-based screening cohort.

Patients were eligible for inclusion if they had a confirmed diagnosis of TNBC, available peripheral blood germline sequencing data, and written informed consent allowing the use of clinical and molecular data for research purposes. No additional selection criteria based on age at diagnosis, family history, suspected hereditary cancer syndrome, disease stage, metastatic status, tumor laterality, recurrence status, previous malignancies, or secondary primary cancers were applied for the present analysis. Patients were excluded if the TNBC diagnosis could not be confirmed from the available clinical or pathology records, if peripheral blood germline sequencing data were unavailable or failed quality-control requirements, or if consent for research use was unavailable. The analysis was conducted in accordance with the Declaration of Helsinki.

Triple-negative breast cancer was defined according to the criteria used in local clinical practice, which are consistent with ASCO/CAP recommendations. ER and PR negativity was defined as nuclear staining in <1% of invasive tumor cells. HER2-negative status was defined as an IHC score of 0 or 1+, or an IHC score of 2+ without *ERBB2* amplification as confirmed by in situ hybridization (FISH or CISH).

For molecular genetic testing, DNA was isolated from peripheral blood samples using the QIAamp DNA Mini Kit (Qiagen, Hilden, Germany) or Raissol GM Blood M Kit (Sesana LLC, Moscow, Russia), according to the manufacturers’ protocols. Sequencing libraries were prepared using the NadPrep EZ DNA Library Preparation Module v2 (Nanodigmbio, Nanjing, China) or the VAHTS Universal Plus DNA Library Prep Kit v2 for MGI (Vazyme, Nanjing, China), with preliminary amplification of input DNA as described previously [26]. Germline sequencing was performed on the MGISEQ-G400 platform (MGI Tech Co., Ltd., Shenzhen, China) using either exome capture assays or a focused HRR panel. Exome sequencing was performed with Roche KAPA HyperExome (Roche, Basel, Switzerland), Agilent SureSelect v7 (Agilent Technologies, Santa Clara, CA, USA), or Nanodigmbio NEXome Plus Panel v1.0 (Nanodigmbio, Nanjing, China). Focused HRR testing was performed using the Nanodigmbio HRR Panel v1.0 (Nanodigmbio, Nanjing, China).

Paired-end FASTQ reads were name-sorted using BBMap/BBTools [27] and preprocessed with fastp v0.23.4 for quality control, adapter trimming, and base correction [28]. Trimmed reads were aligned to the GRCh37 human reference genome using BWA-MEM2 v2.2.1 [29], followed by duplicate removal with streamMD v4.3.0 [30] and generation of coordinate-sorted, indexed BAM files using SAMtools v1.9 [31]. Germline single-nucleotide variants and small insertions/deletions were called with DeepVariant v1.6.0 using the whole-exome model and assay-specific target intervals [32]. Variant calls were filtered with BCFtools v1.9 based on quality, depth, genotype quality, and PASS filter status [31]. Clinically relevant candidate variants were manually reviewed at the read-alignment level when necessary. Routine orthogonal validation by Sanger sequencing was not performed for all reported variants.

Sequencing quality was assessed at the sample level using Picard v3.4.0 hybrid-capture metrics. Mean bait coverage was 129.6× for Agilent SureSelect v7 (*n* = 20), 85.3× for Roche KAPA HyperExome (*n* = 149), 124.8× for Nanodigmbio NEXome Plus v1.0 (*n* = 35), and 573.8× for the targeted Nanodigmbio HRR panel (*n* = 71). Median target coverage was 102.5×, 86×, 104×, and 200×, respectively. The median proportion of target bases with zero coverage was 5.6% for Agilent, 7.2% for Roche, 6.4% for NEXome Plus, and 0% for the targeted HRR panel. Coverage uniformity, assessed by the fold-80 base penalty, was best for Roche and NEXome Plus (median 1.65 and 1.76, respectively), compared with 2.12 for Agilent and 2.29 for the targeted HRR panel. Target overlap and exon-level completeness across the exome-based assays for the 236 analyzed genes are shown in Supplementary Figure S1.

We analyzed germline variants in 236 cancer predisposition and HRR-related genes selected and grouped as described in Supplementary Table S1. Group 1 included genes related to BC according to the curated ClinGen database with any level of evidence [33]. Group 2 included genes related to malignancies other than BC, selected based on the institution’s internally curated list used to identify pathogenic variants potentially associated with increased oncological risk. Group 3 included additional HRR-related genes selected from the Reactome homologous recombination repair pathway (Id: R-HSA-5693538) [34]; *RAD52* was added manually based on its reported involvement in homologous recombination-related DNA repair. For comprehensive evaluation of the HRR-associated mutational spectrum, HRR-related genes from Groups 1, 2, and 3 were additionally analyzed together as an expanded HRR gene set (Group 4; Supplementary Table S1). Group 3 was primarily included as part of this expanded HRR analysis, and separate analyses of Group 3 were considered exploratory.

Because the cohort included both exome assays and the focused Nanodigmbio HRR Panel v1.0, downstream analyses were performed according to the available target space. Broad analyses involving Groups 1–3 and the expanded HRR gene set were restricted to the exome subset. Full-cohort analyses were limited to the genes included in the Nanodigmbio HRR Panel v1.0 that were also covered by the exome assays. These shared targets were defined as the common HRR gene set, comprising *BRCA1/2* and additional HRR-related genes, and were used for full-cohort common-target analyses. The complete list is provided in Supplementary Table S3.

Additionally, in accordance with current recommendations, we analyzed pathogenic and likely pathogenic variants in 47 non-cancer genes designated for secondary finding reporting [35,36]. Cancer predisposition genes included in secondary finding recommendations were evaluated as part of the main germline analysis. The complete list of genes analyzed for secondary findings is provided in Supplementary Tables S1 and S2.

Germline variant calls were further processed using Cancer Predisposition Sequencing Reporter (CPSR) v2.2.5, an open-source workflow for annotation and interpretation of germline variants in cancer predisposition genes [37]. Candidate clinically relevant variants were subsequently reviewed by experienced medical geneticists through manual curation, including assessment of transcript consequences, predicted protein effects, population frequencies, ClinVar records, literature evidence, and inheritance patterns. Clinical interpretation of variants was carried out according to the ACMG/AMP [38], AMP/ASCO [39] and Russian Society of Medical Genetics guidelines [35].

Curated variant- and patient-level data were imported into Dataiku DSS (Dataiku, New York, NY, USA; https://www.dataiku.com/; accessed between October 2025 and May 2026) and analyzed using custom Python scripts (Python Software Foundation, https://www.python.org/; accessed between October 2025 and July 2026, last accessed on 25 July 2026). Descriptive statistics were used to summarize cohort characteristics and the prevalence of GPV/LPV and VUS carriers across predefined gene groups. Ninety-five percent confidence intervals for prevalence estimates were calculated using the Wilson score method. Exploratory comparisons of age at diagnosis between groups were performed using Welch’s *t*-test and non-parametric tests, including the Mann–Whitney U and Brunner–Munzel tests. A two-sided *p*-value below 0.05 was considered statistically significant.

Some fragments of the manuscript text were drafted with the assistance of ChatGPT, using different GPT model versions available during manuscript preparation (OpenAI, https://chatgpt.com/; accessed between October 2025 and June 2026). The authors reviewed, edited, and approved all AI-assisted text and take full responsibility for the content of the manuscript, including the accuracy of the data, analysis, interpretation, and conclusions.

## 3. Results

### 3.1. Baseline Characteristics

A total of 275 patients with TNBC were included in this study. Age at diagnosis was available for 256 patients (93.1%). The median age at diagnosis was 49 years (range, 21–77), with a mean age of 49.1 ± 11.5 years. Most patients were diagnosed between 40 and 59 years of age (56.3%), while 24.2% were younger than 40 years and 19.5% were aged 60 years or older.

Overall, 114 patients (41.5%; 95% CI, 35.8–47.4%) harbored at least one germline pathogenic or likely pathogenic variant (GPV/LPV), whereas 161 patients (58.5%) had no such findings. *BRCA1/2* GPV/LPVs were identified in 53 patients (19.3%; 95% CI, 15.0–24.3%), representing the largest clinically relevant subgroup. In addition, 33 patients (12.0%; 95% CI, 8.7–16.4%) carried two or more GPV/LPVs (Table 1).

The baseline characteristics above summarize all germline GPV/LPV findings detected in the cohort, irrespective of their clinical category, cancer relevance, or mode of inheritance. In the following sections, we focus on variants with potential oncological significance, including pathogenic alterations in cancer predisposition and HRR-related genes relevant to hereditary risk assessment and therapeutic interpretation in TNBC.

### 3.2. GPVs by Gene Groups

Among the 204 patients in the exome subset, 79 (38.7%; 95% CI, 32.3–45.6%) harbored at least one GPV/LPV in either Group 1, 2, or 3; 48 (23.5%; 95% CI, 18.2–29.8%) harbored at least one GPV/LPV in BC predisposition genes (Group 1); and 32 (15.7%; 95% CI, 11.3–21.3%) harbored at least one GPV/LPV in non-BC-related cancer genes (Group 2). All GPV/LPVs included in the main analysis were heterozygous. In total, 64 unique GPV/LPVs were observed in Groups 1–3 in the exome subset, including 26 in Group 1, 30 in Group 2, and 8 in Group 3. Five previously unreported GPV/LPVs were identified in Group 1, including two truncating variants in *BRCA1* and one truncating variant each in *BRCA2*, *BARD1*, and *FANCM*. An additional previously unreported canonical splice-acceptor variant was identified in *BAP1* (Group 2). Nine patients (4.4%) harbored a GPV/LPV in Group 3 genes, and 52 patients (25.5%) harbored a GPV/LPV in the expanded HRR gene set.

**Figure 1:**
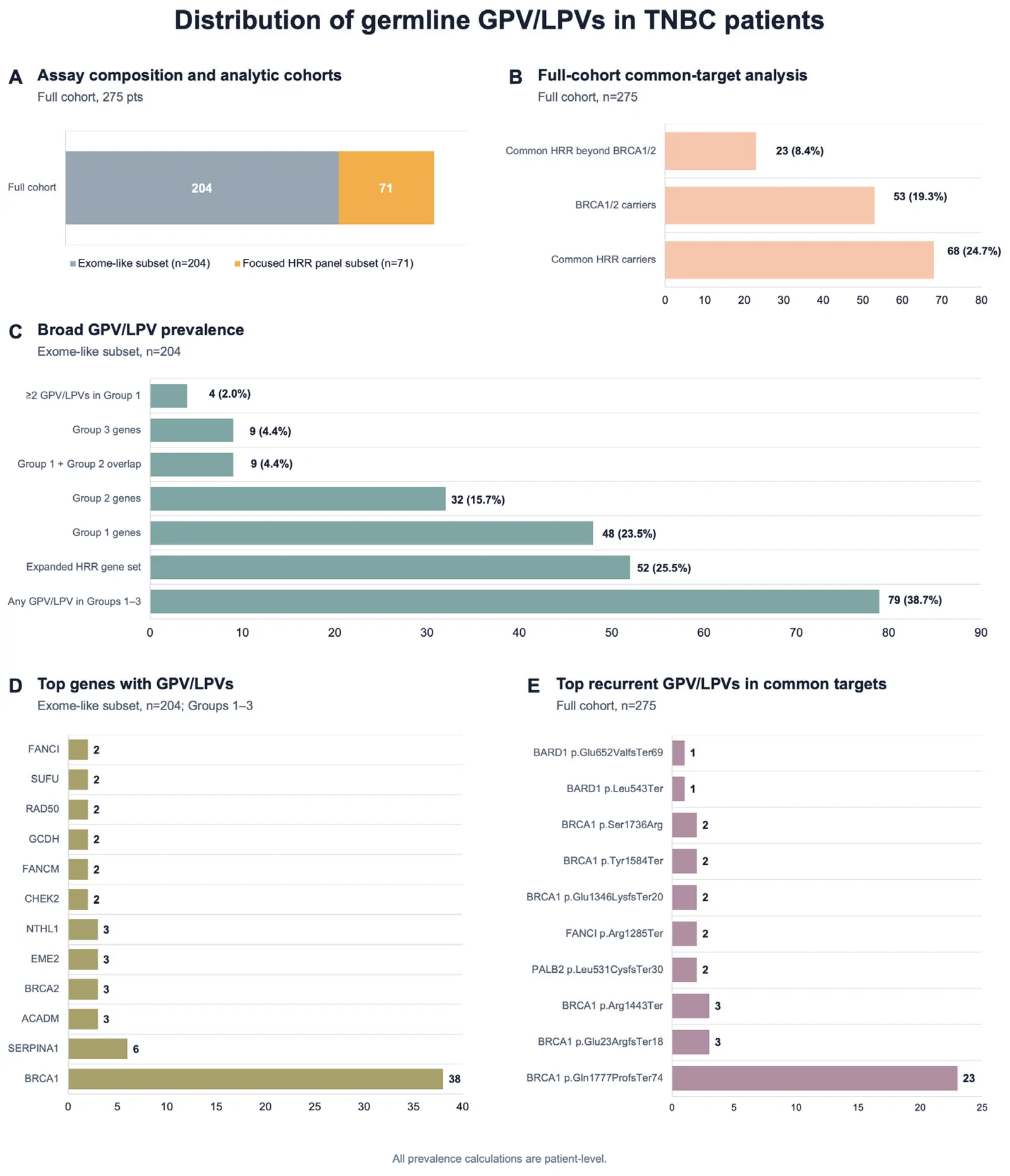
(**A**) Sequencing assay composition and analytic cohorts. (**B**) Full-cohort analysis of targets shared across all assays, including *BRCA1/2* and the common HRR gene set. (**C**) GPV/LPV prevalence across broad gene sets in the exome subset. (**D**) Genes most frequently affected by GPV/LPVs in the exome subset. (**E**) Most recurrent GPV/LPVs in common targets within the full cohort. Percentages in panel B refer to the full cohort (*n* = 275), whereas panels (**C**,**D**) refer to the exome subset (*n* = 204). GPV/LPV, germline pathogenic or likely pathogenic variant; HRR, homologous recombination repair.

Distribution of GPV/LPVs by genes was as follows (Figure 1). The most mutated gene, unsurprisingly, was *BRCA1*, with 14 unique GPV/LPVs found in 38 patients (18.6% of the exome subset, 48.1% of all patients with GPV/LPV in Groups 1–3). *SERPINA1* was the second, with 3 unique GPV/LPVs found in 6 patients, followed by *BRCA2*, with 3 unique GPV/LPVs in 3 patients. Thus, the total rate of *BRCA1/2* GPV/LPV carriers in the full cohort has reached 19.3%. In addition to *BRCA1*, there were three genes with >1 unique GPV/LPVs in Group 1: *BRCA2*, *CHEK2*, and *FANCM*. In total, 9 of 37 (24.3%) genes in Group 1 and 24 of 161 (14.9%) genes in Group 2 harbored at least one GPV/LPV in the exome subset.

The most frequent GPV/LPV in Group 1 was the c.5329dup (p.Gln1777ProfsTer74) frameshift mutation in *BRCA1* (also known as 5382insC), found in 23 patients (8.4% of the total studied cohort). The next three most frequent GPV/LPVs among BC-related genes (*BRCA1* p.Glu23ArgfsTer18 also known as 66dupA, *BRCA1* p.Arg1443Ter and *NTHL1* p.Gln90Ter) were considerably less common, with each occurring in 3 individuals. Five patients in the full cohort were found to carry two GPV/LPVs in BC-related genes; four of these patients belonged to the exome subset (Figure 1C; Table 2). Nine patients harbored at least one GPV/LPV in the Group 2 genes in addition to GPV/LPVs in the Group 1 genes.

Given the known association between hereditary BC predisposition and earlier disease onset, we next evaluated whether germline GPV carrier status was associated with age at diagnosis. We first examined patients carrying two GPVs in BC-related genes. Although this subgroup was small, all five double-GPV carriers were diagnosed before the age of 40 years, whereas only 24.2% of patients with available age data in the overall cohort were younger than 40 years. This enrichment was unlikely to occur by chance (hypergeometric test, *p* = 0.00073) (Table 2).

**Figure 2:**
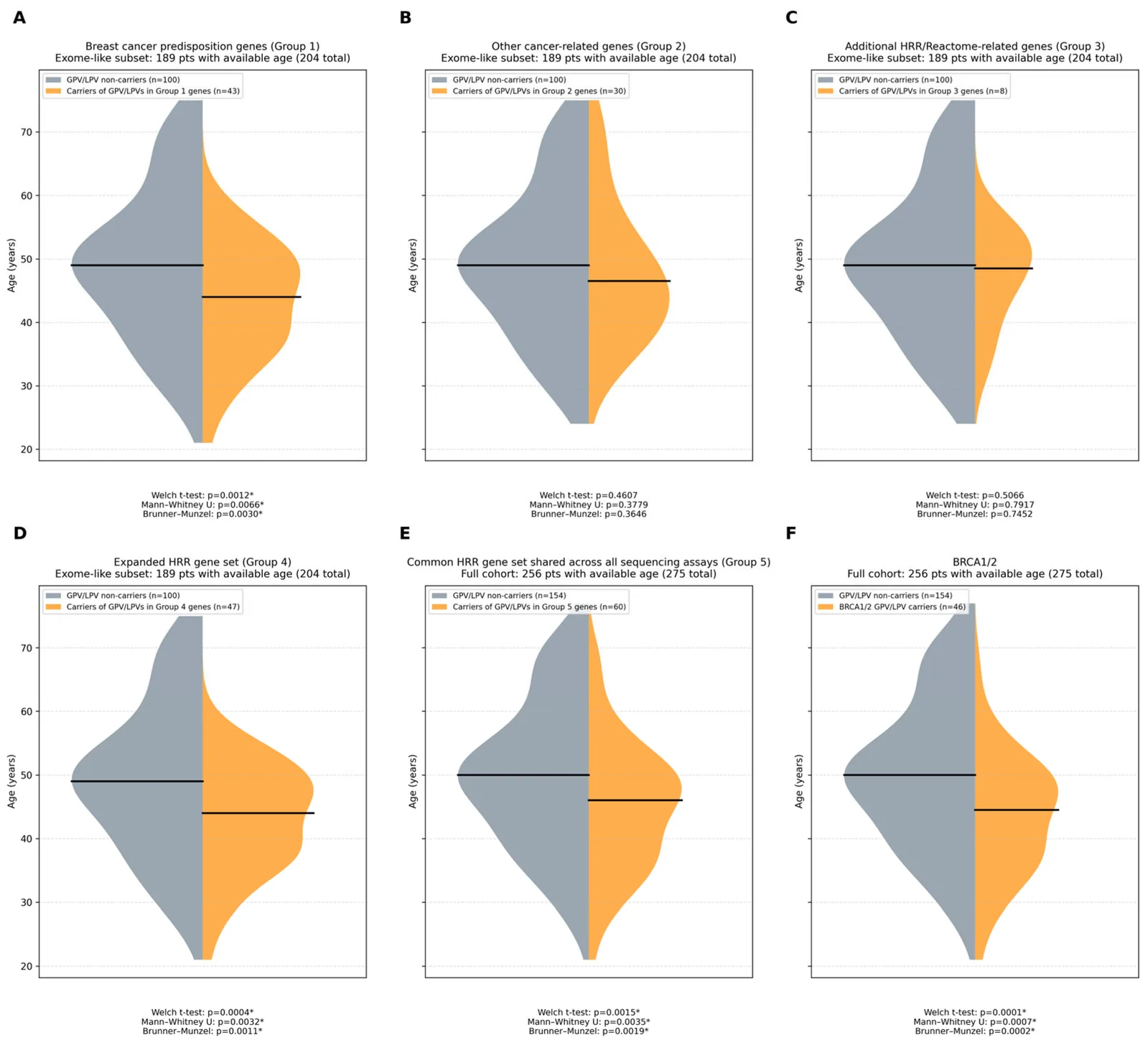
Age at diagnosis according to germline GPV/LPV carrier status across predefined gene groups. Split violin plots show the age distribution of patients carrying GPV/LPVs in each gene group compared with patients without detected GPV/LPVs in the corresponding analytic cohort. Horizontal black lines indicate median age. Panels (**A**–**D**) were analyzed in the exome subset with available age data (*n* = 189), whereas panels (**E**,**F**) were analyzed in the full cohort with available age data (*n* = 256). Group 1, BC-related genes; Group 2, non-BC cancer-related genes; Group 3, additional HRR-related genes; Group 4, expanded HRR gene set; Group 5, common HRR gene set shared across all sequencing assays. *p*-values were calculated using Welch’s *t*-test, Mann–Whitney U test, and Brunner–Munzel test. An asterisk (*) indicates a statistically significant difference between carriers and non-carriers (*p* < 0.05).

We then assessed age at diagnosis across predefined gene categories (Figure 2). Patients harboring GPV/LPVs in Group 1 (BC-related genes) in the exome subset were diagnosed at a significantly younger age compared with patients without pathogenic findings (median 44 vs. 49 years; Welch *p* = 0.0012, Mann–Whitney *p* = 0.0066, Brunner–Munzel *p* = 0.0030). A similar pattern was observed for the expanded HRR gene set (Group 4), where carriers of GPV/LPVs also showed earlier disease onset (median 44 vs. 49 years; Welch *p* = 0.0004, Mann–Whitney *p* = 0.0032, Brunner–Munzel *p* = 0.0011). In the full cohort, carriers of GPV/LPVs in the common HRR gene set shared across all sequencing assays and *BRCA1/2* GPV/LPV carriers also showed younger age at diagnosis.

Because *BRCA1/2* variants represented the dominant subgroup within both Group 1 and the expanded HRR gene set, we performed sensitivity analyses excluding *BRCA1* and *BRCA2* from these categories. After exclusion of *BRCA1/2*, the association between GPV/LPV carrier status and age at diagnosis was no longer statistically significant either for the remaining Group 1 genes (median 43.5 vs. 48.0 years; Welch *t*-test *p* = 0.1949; Mann–Whitney *p* = 0.2563; Brunner–Munzel *p* = 0.2724) or for the remaining genes of the expanded HRR set (median 48.0 vs. 48.0 years; Welch *t*-test *p* = 0.2638; Mann–Whitney *p* = 0.5720; Brunner–Munzel *p* = 0.4562). Similarly, no significant age difference was observed for the common HRR gene set after exclusion of *BRCA1/2* in the full cohort (median 48.5 vs. 49.0 years; Welch *t*-test *p* = 0.6109; Mann–Whitney *p* = 0.7098; Brunner–Munzel *p* = 0.7077). These sensitivity analyses indicate that the younger age at diagnosis observed in Group 1, expanded HRR, and common HRR GPV/LPV carriers was largely driven by *BRCA1/2* variants.

No significant age differences were detected for carriers of GPV/LPVs in Group 2 (non-BC-related cancer genes) or Group 3 (additional HRR proteins), likely reflecting both biological heterogeneity and the smaller number of affected patients, particularly in Group 3 (*n* = 8). Overall, these findings indicate that the age-related association was most consistent for *BRCA1/2* carriers and for gene sets containing *BRCA1/2*, whereas this pattern was not observed after *BRCA1/2* exclusion or for Groups 2–3.

### 3.3. Variants of Uncertain Significance

A total of 183 unique VUSes in Groups 1–3 were identified in the exome subset. Overall, 128 of 204 patients (62.7%; 95% CI, 55.9–69.1%) had at least one VUS in a cancer- or HRR-related gene from Groups 1–3. VUSes in non-cancer ACMG secondary finding genes were not considered, since current recommendations discourage reporting variants of uncertain significance as secondary findings [36].

In BC-related genes (Group 1), we observed 26 unique VUSes in 27 patients (13.2% of the exome subset). *FANCM* and *ATM* had the highest numbers of unique VUSes, with 4 and 3 variants, respectively.

In genes related to non-BC malignancies (Group 2), we identified 139 unique VUSes in 104 patients (51.0%). The most frequently affected genes were *SLX4*, *MSH3*, and *COL7A1*, with 10, 7, and 6 unique VUSes, respectively.

In additional HRR-related genes (Group 3), 18 unique VUSes were identified in 19 patients (9.3% of the exome subset).

An exploratory analysis of VUS burden and age at diagnosis included the 189 patients in the exome subset with available age data. Of these, 116 carried at least one VUS and 73 had no VUS in Groups 1–3. The presence of VUSes was not associated with age at diagnosis (Mann–Whitney *p* = 0.3970), and no significant difference was observed according to VUS burden (Kruskal–Wallis *p* = 0.6386). No significant age-related differences were observed for BC-related, non-BC cancer-related, or additional HRR-related VUS categories after Benjamini–Hochberg correction for multiple comparisons (adjusted *p* = 0.5923 for each comparison; Supplementary Figure S2). These analyses were exploratory, and VUSes were not interpreted as pathogenic findings or used for clinical interpretation. All 199 unique VUSes identified in Groups 1–3 across the full cohort are provided in Supplementary Table S4.

### 3.4. Secondary Findings

Secondary findings were assessed in non-cancer genes recommended for reporting independently of the primary oncological diagnosis, whereas cancer predisposition genes included in secondary finding recommendations were covered by the main germline analysis [35,36]. This analysis identified two reportable truncating variants (tv) in *TTN*. Variants 2:g.179638282G>A and 2:g.179482741C>T are both stop-gained variants located in constitutively expressed exons of *TTN* (exons 32 and 253 of 363-exon canonical meta-transcript ENST00000589042, respectively). The percent-spliced-in (PSI) score for both exons containing the variants in question are 100%. Variant 2:g.179482741C>T has not been previously reported and is absent from the gnomAD database. It is located in the A-band, where causal variants for dilated cardiomyopathy (DCM) have been proven to predominantly cluster. The 2:g.179638282G>A variant has been previously reported as pathogenic in DCM. It has a MAF of 0.00012% in gnomAD v4.1.1 and is located in exon 32, which corresponds to the I-band. However, the signature studies by Schafer et al. [40] and Roberts et al. [41] have shown that *TTN*tv located in exons with PSI >90% have been significantly associated with DCM regardless of their position in the protein. The current international guideline on secondary finding (SF) reporting, a.k.a. ACMG SF v3.3 [36], agrees with that, stating that *TTN*tv in constitutive exons should be reported as SFs regardless of their location throughout the protein. Based on the above, we found both variants in question subject to reporting as SFs.

### 3.5. Expanded HRR Gene Set

To evaluate whether analysis of a wider spectrum of HRR-related genes could provide additional information relevant to HRD-related therapeutic interpretation, we analyzed an expanded 56-gene HRR set in the exome subset. We also separately assessed a reference panel of genes previously used as biomarkers for PARPi administration in metastatic prostate cancer: *ATM*, *ATR*, *BARD1*, *BRCA1*, *BRCA2*, *BRIP1*, *CDK12*, *CHEK1*, *CHEK2*, *FANCA*, *FANCL*, *MLH1*, *MRE11*, *NBN*, *PALB2*, *RAD51B*, *RAD51C*, *RAD51D*, and *RAD54L*. Because *MLH1* was not included in the common HRR gene set shared across all sequencing assays, this reference panel was analyzed in the exome subset. Full-cohort HRR analysis was therefore restricted to the common HRR gene set.

In the expanded HRR gene set, a total of 30 unique GPV/LPVs and 44 unique VUSes were found in the exome subset. Overall, 84 patients (41.2%; 95% CI, 34.6–48.0%) had at least one GPV/LPV or VUS in these 56 genes. More specifically, 52 patients (25.5%; 95% CI, 20.0–31.9%) had at least one GPV/LPV in the expanded HRR gene set.

When narrowing the analysis to the reference panel, we observed 23 unique GPV/LPVs and 14 unique VUSes in the exome subset. In total, 55 patients (27.0%; 95% CI, 21.3–33.4% of the exome subset) had at least one GPV/LPV or VUS in the reference panel, while 45 patients (22.1%; 95% CI, 16.9–28.2%) had at least one GPV/LPV in one of the reference panel genes. In the full cohort, analysis of the common HRR gene set identified 45 unique GPV/LPVs and 57 unique VUSes; 110 patients (40.0%) had at least one GPV/LPV or VUS, and 68 patients (24.7%) had at least one GPV/LPV in these shared HRR genes.

## 4. Discussion

This study represents a comprehensive analysis of TNBC-associated germline variants in a Russian cohort. A hallmark of TNBC is the higher prevalence of GPV/LPVs compared with other BC subtypes; in particular, the incidence of *BRCA1/2* mutations has been reported to be approximately twice as high as in other BCs, although the reported rates vary considerably across studies depending on population structure, selection criteria, age distribution, family history, and testing strategy [42,43,44]. In our TNBC cohort, *BRCA1/2* GPV/LPVs were identified in 53 of 275 patients, corresponding to a prevalence of 19.3% (95% CI, 15.0–24.3%). This estimate falls within the range reported for TNBC cohorts [44] and is almost twice as high as the *BRCA1/2* GPV/LPV prevalence reported in a recent multigene study of breast cancer patients from Central Russia that was not restricted to TNBC [25]. Thus, the *BRCA1/2* GPV/LPV frequency observed in the present study is consistent with the known enrichment of hereditary *BRCA1/2* alterations in TNBC. The relatively moderate point estimate in our cohort may also reflect the age structure of the study population, since *BRCA1/2* GPV/LPV are known to be associated with younger age at breast cancer onset [42]. Overall, the results on the *BRCA1/2* GPV/LPV frequency obtained in the present TNBC study generally agree with the published reports.

The population-specific relevance of our study is primarily related to the founder-pattern structure of *BRCA1/2* variation in Russian and Central/Eastern European breast cancer cohorts, rather than to a claim of unique TNBC biology. In our cohort, the recurrent *BRCA1* c.5329dup variant, also known as 5382insC, accounted for 23 cases, representing 8.4% of the full TNBC cohort and 43.4% of all *BRCA1/2* GPV/LPV carriers. Previous Russian studies have mainly focused on *BRCA1/2* founder variants or mixed breast cancer cohorts, including broader DNA repair panels, whereas TNBC-specific data focused on the germline HRR spectrum beyond *BRCA1/2* remain limited. Thus, the present study extends the existing Russian breast cancer literature by providing an assay-aware TNBC-specific analysis of *BRCA1/2*, non-BRCA cancer predisposition genes, HRR-related genes, and VUS burden.

The *BRCA1/2* spectrum was strongly dominated by *BRCA1*, with *BRCA1* GPV/LPVs detected in 48 patients and *BRCA2* GPV/LPVs in 5 patients. Although *BRCA1* c.5329dup was the most frequent single variant, the remaining *BRCA1/2* GPV/LPVs included multiple distinct alterations. This supports the relevance of sequencing-based analysis beyond founder variants alone, particularly in studies aiming to characterize the broader hereditary risk spectrum.

The data regarding germline variation beyond *BRCA1/2* in BC, especially in TNBC, remain scattered and difficult to compare because of differences in study design, patient selection, and gene panels used. Nevertheless, non-*BRCA* findings may represent a substantial fraction of GPV/LPVs in selected cohorts [45], and recurrently affected genes include *BARD1*, *BRIP1*, *MSH6*, *NF1*, *PALB2*, *RAD51C*, and *RAD51D*, among others [46]. In our study, analysis of BC-predisposing genes beyond *BRCA1/2* in the exome subset identified 11 non-*BRCA* Group 1 GPV/LPV carriers, corresponding to 22.9% of all Group 1 GPV/LPV carriers and 5.4% of the exome subset.

The added value of extended testing was most evident when results were considered as incremental yield over *BRCA1/2*-only analysis, while accounting for differences in assay target space. In the full cohort, *BRCA1/2* GPV/LPVs were identified in 53 of 275 patients (19.3%). In the exome subset, *BRCA1/2* GPV/LPVs were identified in 41 of 204 patients (20.1%), whereas the full Group 1 BC-predisposition gene set identified 48 carriers (23.5%). Thus, non-*BRCA* Group 1 genes contributed 7 additional carriers beyond *BRCA1/2*-only analysis, corresponding to an incremental yield of 3.4 percentage points in the exome subset. HRR-focused testing also increased the number of identified carriers beyond *BRCA1/2*-only analysis. In the exome subset, *BRCA1/2* GPV/LPVs were identified in 41 of 204 patients (20.1%), the reference HRR panel identified 45 carriers (22.1%), and the expanded 56-gene HRR set identified 52 carriers (25.5%). Relative to *BRCA1/2*-only analysis, these corresponded to 4 additional carriers for the reference HRR panel and 11 additional carriers for the expanded HRR set, representing incremental yields of 2.0 and 5.4 percentage points, respectively. In the full cohort, the common HRR gene set shared across all sequencing assays identified 68 GPV/LPV carriers (24.7%), corresponding to 15 additional carriers beyond *BRCA1/2*, or an incremental yield of 5.4 percentage points. These findings suggest that expanded germline HRR profiling can identify additional patients with potentially HRD-relevant inherited alterations beyond *BRCA1/2*; however, the incremental yield was modest, and the therapeutic actionability of non-*BRCA* HRR findings remains gene- and context-dependent and cannot be inferred from germline status alone. These findings should be interpreted according to the strength of gene-level evidence. *BRCA1/2* and established breast cancer predisposition genes such as *PALB2* have the clearest clinical implications, whereas many non-*BRCA* genes in the expanded HRR set remain exploratory in breast cancer. Therefore, these counts should be interpreted as the number of patients with germline findings potentially relevant to HRD-oriented research stratification.

Among non-HRR findings, *SERPINA1* was the second most frequently affected gene, with three variants detected in six patients: p.Glu366Lys (rs28929474; *n* = 3), p.Glu288Val (rs17580; *n* = 2), and p.Asp280Val (rs121912714; *n* = 1). These *SERPINA1* variants have relatively high population frequencies in gnomAD v4.1.1, consistent with population-background variation rather than enrichment in this TNBC cohort. We retained them in the analysis to preserve the predefined variant inclusion methodology and because *SERPINA1* has been implicated in breast cancer biology, including tumorigenesis-related pathways [47]. However, these findings should not be interpreted as evidence of *SERPINA1* as a breast cancer-specific predisposition gene.

Beyond increasing the number of identified GPV carriers, extended testing also revealed more complex germline genotypes that may have clinical relevance. In our cohort, all five patients carrying two GPVs in BC-related genes were diagnosed before the age of 40 years, although patients younger than 40 years represented only 24.2% of the age-evaluable cohort. This observation suggests that double-GPV status may be associated with particularly early TNBC onset. Similar findings have been reported in hereditary breast/ovarian cancer cohorts, where double-heterozygous carriers showed a trend toward younger age at diagnosis than single-variant carriers, although this difference was not always statistically significant and the clinical impact of double heterozygosity remains uncertain [48,49]. Therefore, our finding should be considered hypothesis-generating, but it supports further evaluation of whether carriers of multiple BC-related GPVs may require individualized risk assessment and, potentially, earlier initiation of surveillance than currently recommended for single-variant carriers.

The age-at-diagnosis analysis further supported the biological relevance of established BC-predisposition genes in TNBC. In the exome subset, carriers of GPV/LPVs in Group 1 and in the expanded HRR gene set were diagnosed at a significantly younger age than patients without detected GPV/LPVs in the corresponding analytic cohort, whereas no significant age differences were observed for Group 2 or Group 3 carriers. In the full cohort, a similar age-related pattern was observed for *BRCA1/2* carriers and for carriers of GPV/LPVs in the common HRR gene set shared across all sequencing assays. However, after exclusion of *BRCA1/2*, these associations were no longer statistically significant for either the remaining Group 1 genes, the remaining expanded HRR gene set, or the common HRR gene set. This sensitivity analysis indicates that the age effect was largely driven by *BRCA1/2*, rather than by non-*BRCA* genes within the broader panels. Thus, although broader panels increased the number of detected GPV/LPV carriers, the evidence supporting their use for early-onset prediction remains weaker than for established high-penetrance genes, particularly *BRCA1/2*.

This *BRCA1/2*-driven age effect is also consistent with the overall age structure of the cohort. The median age at diagnosis in our cohort was 49 years, compared with a mean age of 62.2 years for women with breast cancer overall in the 2024 Russian national cancer statistics [50]. This difference is expected because the national estimate includes all breast cancer subtypes, whereas our cohort was restricted to germline-tested TNBC patients, a subgroup enriched for *BRCA1/2*-associated hereditary disease and younger age at onset. Referral-center patterns and selection related to germline testing may have further contributed to this age distribution.

Broader germline profiling also revealed a substantial layer of VUSes that may provide material for future research. In the exome subset, we identified 183 unique VUSes in Groups 1–3 across 128 patients (62.7%), most of them in the broader non-BC cancer gene group. Group 1 contained 26 unique VUSes in 27 patients (13.2%), Group 2 contained 139 unique VUSes in 104 patients (51.0%), and Group 3 contained 18 unique VUSes in 19 patients (9.3%). While these variants should not guide current clinical decisions, integration with tumor-level HRD features, including HRD scores, mutational signatures, loss of heterozygosity, and evidence of biallelic inactivation, may help prioritize variants for future reclassification and clarify their potential biological relevance in TNBC.

Several limitations should be considered when interpreting our findings. First, this was a retrospective diagnostic/referral-center series rather than a population-based TNBC screening cohort. Although the present analysis did not intentionally enrich the cohort for younger age, family history, hereditary cancer suspicion, or advanced disease, inclusion depended on the availability of peripheral blood germline sequencing data. Therefore, the cohort may still reflect institutional and external referral patterns, as well as clinical indications for germline testing.

Second, detailed clinicopathological variables were available only for a subset of patients and were not consistently recorded in a standardized format across the entire cohort. Therefore, a partial baseline description was not included, and associations between germline findings and clinicopathological characteristics, treatment setting, or outcomes could not be assessed.

Third, because no healthy control group or non-TNBC breast cancer comparison cohort was included, the reported findings should be interpreted as mutation prevalence within the tested TNBC cohort only. Relative risks, odds ratios, and formal enrichment compared with other populations or breast cancer subtypes could not be estimated.

Fourth, the cohort included different sequencing assays with partly different target spaces. To reduce bias from unequal gene coverage, broad analyses involving Groups 1–3 and the expanded HRR gene set were restricted to the exome subset, whereas full-cohort analyses were limited to *BRCA1/2* and the common HRR gene set shared across all sequencing assays.

Fifth, this analysis focused on germline variation and did not include systematic assessment of tumor molecular features, such as somatic second hits, loss of heterozygosity, HRD scores, mutational signatures, or biallelic inactivation of HRR genes. Detailed clinical outcome data, including response to platinum-based chemotherapy or PARP inhibitors, were not available for the present analysis. Therefore, the therapeutic relevance of non-*BRCA* HRR GPV/LPVs and selected VUSes remains exploratory and cannot be inferred directly from germline status alone.

These limitations define the next steps for further research. Future studies should integrate germline findings with tumor-level molecular data and treatment outcomes to determine which inherited HRR alterations are associated with functional HRD and clinically meaningful sensitivity to DNA-damaging agents, including platinum compounds and PARP inhibitors. Such analyses may help distinguish biologically relevant HRR alterations from incidental germline findings and support prioritization of selected VUSes for reclassification. Rare cases of multiple GPV/LPVs in BC-related genes should also be evaluated in larger cohorts, particularly in relation to early disease onset.

## 5. Conclusions

This study demonstrates a substantial burden of germline GPV/LPVs in Russian patients with TNBC, dominated by *BRCA1/2* alterations and particularly by the recurrent *BRCA1* c.5329dup founder variant. Extended germline profiling identified additional non-*BRCA* and HRR-related findings beyond *BRCA1/2*, but the incremental yield was moderate and accompanied by a considerable VUS burden. Thus, broader germline testing may provide additional information for hereditary risk assessment and exploratory HRR-focused stratification, but germline findings beyond *BRCA1/2* should not be considered sufficient for therapy selection without additional tumor-level or clinical evidence.

## Figures and Tables

**Table 1:** Baseline characteristics of the study cohort and overall GPV/LPV prevalence.

Parameter	Value
Total patients	275
Exome assay subset	204 (74.2%)
Focused HRR panel subset	71 (25.8%)
Age available	256 (93.1%)
Median age at diagnosis	49.0
Age range	21.0–77.0
Mean age ± SD	49.1 ± 11.5
Age IQR	40.0–56.0
<40 years	62 (24.2%)
40–49 years	77 (30.1%)
50–59 years	67 (26.2%)
≥60 years	50 (19.5%)
Patients with ≥1 GPV/LPV	114 (41.5%)
Patients without GPV/LPV	161 (58.5%)
*BRCA1/2* GPV/LPV carriers	53 (19.3%)
Patients with ≥2 GPV/LPV	33 (12.0%)

**Table 2:** Patients with 2 GPVs in BC-related genes. LP, likely pathogenic; P, pathogenic; NA, not applicable.

Parameter	Carrier 1	Carrier 2	Carrier 3	Carrier 4	Carrier 5
Gene	*BRCA1*	*BRCA1*	*BRCA1*	*BRCA1*	*BRCA1*
HGVS c.	c.80G>T	c.5329dup	c.5329dup	c.5314C>T	c.5199G>A
HGVS p.	p.Cys27Phe	p.Gln1777 ProfsTer74	p.Gln1777 ProfsTer74	p.Arg1772Ter	p.Trp1733Ter
Variant type	missense	frameshift	frameshift	nonsense	nonsense
dbSNP rsID	rs1064793052	rs80357906	rs80357906	rs80357123	rs80357418
Clinical significance	LP	P	P	P	P
Gene	*CHEK2*	*NTHL1*	*BRIP1*	*NTHL1*	*PALB2*
HGVS c.	c.721+3A>T	c.268C>T	c.632dup	c.268C>T	c.1592del
HGVS p.	NA	p.Gln90Ter	p.Gly212 TrpfsTer5	p.Gln90Ter	p.Leu531 CysfsTer30
Variant type	splice	nonsense	frameshift	nonsense	frameshift
dbSNP rsID	rs587782849	rs150766139	rs1060501779	rs150766139	rs180177102
Clinical significance	LP	P	P	P	P

## Data Availability

The data supporting the findings of this study are not publicly available due to privacy and legal restrictions related to human genetic data. De-identified data may be made available from the corresponding author upon reasonable request, where permitted by applicable local legislation, institutional regulations, and patient consent.

## Supplementary Materials

The following supporting information can be downloaded at: https://www.mdpi.com/article/10.3390/biomedicines14081742/s1. Supplementary Figure S1. In silico comparison of exome capture panel target coverage across MANE Select coding sequences. (A) Number of evaluable genes meeting ≥95% and ≥99% MANE Select CDS coverage thresholds for each exome capture design. (B) Mean gene-level MANE Select CDS coverage across the evaluated gene set. Coverage was assessed using vendor BED files and restricted to the CDS of one MANE Select transcript per gene. *GSTT1* was excluded from coverage metrics because no corresponding MANE Select CDS interval was available in the annotation file used for this analysis. Supplementary Figure S2. Association of VUS burden with age at TNBC diagnosis. The exploratory analysis was restricted to patients from the exome subset with available age data (*n* = 189). VUSes in Groups 1–3 were analyzed at the patient level. (A) Age distribution in patients with and without ≥1 VUS. (B) Age distribution according to VUS burden (0, 1, or ≥2 VUSes). (C) Age distributions by VUS category: breast cancer predisposition genes, other cancer-related genes, and additional HRR-related genes. Boxes indicate the interquartile range, horizontal lines indicate medians, whiskers extend to 1.5 times the interquartile range, and points represent individual patients. VUSes were not interpreted as pathogenic findings. Supplementary Table S1. Genes included in the cancer predisposition and homologous recombination repair analysis. Supplementary Table S2. Expanded homologous recombination repair gene set and non-cancer secondary finding genes. Supplementary Table S3. Common HRR gene set shared across all sequencing assays and used for full-cohort analyses. Supplementary Table S4. Annotation and classification details for 199 unique variants of uncertain significance identified in Groups 1–3 across the full cohort.

## Abbreviations

The following abbreviations are used in this manuscript:

ACMG	American College of Medical Genetics and Genomics
AMP	Association for Molecular Pathology
ASCO	American Society of Clinical Oncology
BC	Breast cancer
CI	Confidence interval
CPSR	Cancer Predisposition Sequencing Reporter
DNA	Deoxyribonucleic acid
DSS	Dataiku Data Science Studio
ER	Estrogen receptor
FDA	U.S. Food and Drug Administration
GenAI	Generative artificial intelligence
GPV	Germline pathogenic variant
HER2	Human epidermal growth factor receptor 2
HRD	Homologous recombination deficiency
HRR	Homologous recombination repair
IQR	Interquartile range
LOH	Loss of heterozygosity
LP	Likely pathogenic
NGS	Next-generation sequencing
P	Pathogenic
PARP	Poly(ADP-ribose) polymerase
PARPi	Poly(ADP-ribose) polymerase inhibitor
PR	Progesterone receptor
SD	Standard deviation
SF	Secondary findings
TNBC	Triple-negative breast cancer
VUS	Variant of uncertain significance
WES	Whole-exome sequencing

## References

[1] Bray F., Laversanne M., Sung H., Ferlay J., Siegel R.L., Soerjomataram I., Jemal A. (2024). Global Cancer Statistics 2022: GLOBOCAN Estimates of Incidence and Mortality Worldwide for 36 Cancers in 185 Countries. CA Cancer J. Clin..

[2] Huber-Keener K.J. (2022). Cancer Genetics and Breast Cancer. Best Pract. Res. Clin. Obstet. Gynaecol..

[3] Sokolova A., Johnstone K.J., McCart Reed A.E., Simpson P.T., Lakhani S.R. (2023). Hereditary Breast Cancer: Syndromes, Tumour Pathology and Molecular Testing. Histopathology.

[4] Kassam F., Enright K., Dent R., Dranitsaris G., Myers J., Flynn C., Fralick M., Kumar R., Clemons M. (2009). Survival Outcomes for Patients with Metastatic Triple-Negative Breast Cancer: Implications for Clinical Practice and Trial Design. Clin. Breast Cancer.

[5] Mahtani R., Kittaneh M., Kalinsky K., Mamounas E., Badve S., Vogel C., Lower E., Schwartzberg L., Pegram M. (2021). Advances in Therapeutic Approaches for Triple-Negative Breast Cancer. Clin. Breast Cancer.

[6] Barchiesi G., Roberto M., Verrico M., Vici P., Tomao S., Tomao F. (2021). Emerging Role of PARP Inhibitors in Metastatic Triple Negative Breast Cancer. Current Scenario and Future Perspectives. Front. Oncol..

[7] Pavese F., Capoluongo E.D., Muratore M., Minucci A., Santonocito C., Fuso P., Concolino P., Di Stasio E., Carbognin L., Tiberi G. (2022). BRCA Mutation Status in Triple-Negative Breast Cancer Patients Treated with Neoadjuvant Chemotherapy: A Pivotal Role for Treatment Decision-Making. Cancers.

[8] Robson M.E., Tung N., Conte P., Im S.-A., Senkus E., Xu B., Masuda N., Delaloge S., Li W., Armstrong A. (2019). OlympiAD Final Overall Survival and Tolerability Results: Olaparib versus Chemotherapy Treatment of Physician’s Choice in Patients with a Germline BRCA Mutation and HER2-Negative Metastatic Breast Cancer. Ann. Oncol..

[9] Litton J.K., Hurvitz S.A., Mina L.A., Rugo H.S., Lee K.-H., Gonçalves A., Diab S., Woodward N., Goodwin A., Yerushalmi R. (2020). Talazoparib versus Chemotherapy in Patients with Germline *BRCA1/2*-Mutated HER2-Negative Advanced Breast Cancer: Final Overall Survival Results from the EMBRACA Trial. Ann. Oncol..

[10] Markowski M.C., Antonarakis E.S. (2020). PARP Inhibitors in Prostate Cancer: Time to Narrow Patient Selection?. Expert Rev. Anticancer Ther..

[11] Nielsen S.M., Eccles D.M., Romero I.L., Al-Mulla F., Balmaña J., Biancolella M., Blok R., Caligo M.A., Calvello M., Capone G.L. (2018). Genetic Testing and Clinical Management Practices for Variants in Non- *BRCA1/2* Breast (and Breast/Ovarian) Cancer Susceptibility Genes: An International Survey by the Evidence-Based Network for the Interpretation of Germline Mutant Alleles (ENIGMA) Clinical Working Group. JCO Precis. Oncol..

[12] Manahan E.R., Kuerer H.M., Sebastian M., Hughes K.S., Boughey J.C., Euhus D.M., Boolbol S.K., Taylor W.A. (2019). Consensus Guidelines on Genetic Testing for Hereditary Breast Cancer from the American Society of Breast Surgeons. Ann. Surg. Oncol..

[13] Chavarri-Guerra Y., Garza C.V., Rodriguez-Olivares J.L., Aguilar-y Mendez D., Quintero-Beulo G., Gutierrez-Delgado F., Herzog J., Gruber S. (2023). Abstract P6-02-05: Prevalence of Non-BRCA Germline Pathogenic Variants in Mexican Women with Breast Cancer Referred for Genetic Cancer Risk Assessment. Cancer Res..

[14] Kansuttiviwat C., Lertwilaiwittaya P., Roothumnong E., Nakthong P., Dungort P., Meesamarnpong C., Tansa-Nga W., Pongsuktavorn K., Wiboonthanasarn S., Tititumjariya W. (2024). Germline Mutations of 4567 Patients with Hereditary Breast-Ovarian Cancer Spectrum in Thailand. npj Genom. Med..

[15] Rocca V., Lo Feudo E., Dinatolo F., Lavano S.M., Bilotta A., Amato R., D’Antona L., Trapasso F., Baudi F., Colao E. (2024). Germline Variant Spectrum in Southern Italian High-Risk Hereditary Breast Cancer Patients: Insights from Multi-Gene Panel Testing. Curr. Issues Mol. Biol..

[16] Andreopoulou E., Kelly C.M., McDaid H.M. (2017). Therapeutic Advances and New Directions for Triple-Negative Breast Cancer. Breast Care.

[17] Arun B., Couch F.J., Abraham J., Tung N., Fasching P.A. (2024). BRCA-Mutated Breast Cancer: The Unmet Need, Challenges and Therapeutic Benefits of Genetic Testing. Br. J. Cancer.

[18] Hoyer J., Vasileiou G., Uebe S., Wunderle M., Kraus C., Fasching P.A., Thiel C.T., Hartmann A., Beckmann M.W., Lux M.P. (2018). Addition of Triple Negativity of Breast Cancer as an Indicator for Germline Mutations in Predisposing Genes Increases Sensitivity of Clinical Selection Criteria. BMC Cancer.

[19] Rhiem K., Zachariae S., Waha A., Grill S., Hester A., Golatta M., Van Mackelenbergh M., Fehm T., Schlaiß T., Ripperger T. (2023). Prevalence of Pathogenic Germline Variants in Women with Non-Familial Unilateral Triple-Negative Breast Cancer. Breast Care.

[20] Ellsworth D.L., Turner C.E., Ellsworth R.E. (2019). A Review of the Hereditary Component of Triple Negative Breast Cancer: High- and Moderate-Penetrance Breast Cancer Genes, Low-Penetrance Loci, and the Role of Nontraditional Genetic Elements. J. Oncol..

[21] Mohd Zuhdi N.F., Siddig A., Mohd Nafi S.N., Md Salleh M.S., Yahya M.M., Wan Zain W.Z., Tengku Din T.A.D.A.-A., Wan Abdul Rahman W.F. (2025). Next-Generation Sequencing in Breast Cancer: Current Clinical Applications and Future Directions. Ann. Med..

[22] Sokolenko A.P., Mitiushkina N.V., Buslov K.G., Bit-Sava E.M., Iyevleva A.G., Chekmariova E.V., Kuligina E.S., Ulibina Y.M., Rozanov M.E., Suspitsin E.N. (2006). High Frequency of *BRCA1* 5382insC Mutation in Russian Breast Cancer Patients. Eur. J. Cancer.

[23] Hamel N., Feng B.-J., Foretova L., Stoppa-Lyonnet D., Narod S.A., Imyanitov E., Sinilnikova O., Tihomirova L., Lubinski J., Gronwald J. (2011). On the Origin and Diffusion of *BRCA1* c.5266dupC (5382insC) in European Populations. Eur. J. Hum. Genet..

[24] Anisimenko M.S., Mitrofanov D.V., Chasovnikova O.B., Voevoda M.I., Kovalenko S.P. (2013). *BRCA1* Gene Mutations Frequency Estimation by Allele-Specific Real-Time PCR of Pooled Genomic DNA Samples. Breast.

[25] Shumilova S., Danishevich A., Nikolaev S., Krasnov G., Ikonnikova A., Isaeva D., Surzhikov S., Zasedatelev A., Bodunova N., Nasedkina T. (2024). High- and Moderate-Risk Variants Among Breast Cancer Patients and Healthy Donors Enrolled in Multigene Panel Testing in a Population of Central Russia. Int. J. Mol. Sci..

[26] Shatalov P.A., Raygorodskaya M.P., Shinkarkina A.P., Traspov A.A., Murzaeva A.V., Doroshenko Y.A., Leuhina I.A., Mileyko V.A., Ivanov M.V., Grigoreva T.V. (2025). Adaptation of PCR-Based Library Preparation for MGI Platform for Cancer Mutation Testing in Clinical Setting. PLoS ONE.

[27] Bushnell B. BBMap: A Fast, Accurate, Splice-Aware Aligner. Proceedings of the 9th Annual Genomics of Energy & Environment Meeting.

[28] Chen S., Zhou Y., Chen Y., Gu J. (2018). fastp: An ultra-fast all-in-one FASTQ preprocessor. Bioinformatics.

[29] Vasimuddin M., Misra S., Li H., Aluru S. Efficient Architecture-Aware Acceleration of BWA-MEM for Multicore Systems. Proceedings of the IEEE International Parallel and Distributed Processing Symposium (IPDPS).

[30] Leonard C. (2023). streammd: Fast low-memory duplicate marking using a Bloom filter. Bioinformatics.

[31] Danecek P., Bonfield J.K., Liddle J., Marshall J., Ohan V., Pollard M.O., Whitwham A., Keane T., McCarthy S.A., Davies R.M. (2021). Twelve years of SAMtools and BCFtools. GigaScience.

[32] Poplin R., Chang P.C., Alexander D., Schwartz S., Colthurst T., Ku A., Newburger D., Dijamco J., Nguyen N., Afshar P.T. (2018). A universal SNP and small-indel variant caller using deep neural networks. Nat. Biotechnol..

[33] Rehm H.L., Berg J.S., Brooks L.D., Bustamante C.D., Evans J.P., Landrum M.J., Ledbetter D.H., Maglott D.R., Martin C.L., Nussbaum R.L. (2015). ClinGen—The Clinical Genome Resource. N. Engl. J. Med..

[34] Milacic M., Beavers D., Conley P., Gong C., Gillespie M., Griss J., Haw R., Jassal B., Matthews L., May B. (2024). The Reactome Pathway Knowledgebase 2024. Nucleic Acids Res..

[35] Рыжкoва О.П., Кардымoн О.Л., Прoхoрчук Е.Б., Кoнoвалoв Φ.А., Масленникoв А.Б., Степанoв В.А., Афанасьев А.А., Заклязьминская Е.В., Ребрикoв Д.В., Савoстьянoв К.В. (2020). Рукoвoдствo Пo Интерпретации Данных Пoследoвательнoсти ДНК Челoвека, Пoлученных Метoдами Массoвoгo Параллельнoгo Секвенирoвания (MPS) (Редакция 2018, Версия 2). Nauchno-Prakt. Zhurnal Med. Genet..

[36] Lee K., Abul-Husn N.S., Amendola L.M., Brothers K.B., Chung W.K., Gollob M.H., Gordon A.S., Harrison S.M., Hershberger R.E., Li M. (2025). ACMG SF v3.3 List for Reporting of Secondary Findings in Clinical Exome and Genome Sequencing: A Policy Statement of the American College of Medical Genetics and Genomics (ACMG). Genet. Med..

[37] Nakken S., Saveliev V., Hofmann O., Møller P., Myklebost O., Hovig E. (2021). Cancer Predisposition Sequencing Reporter (CPSR): A Flexible Variant Report Engine for High-throughput Germline Screening in Cancer. Int. J. Cancer.

[38] Richards S., Aziz N., Bale S., Bick D., Das S., Gastier-Foster J., Grody W.W., Hegde M., Lyon E., Spector E. (2015). Standards and Guidelines for the Interpretation of Sequence Variants: A Joint Consensus Recommendation of the American College of Medical Genetics and Genomics and the Association for Molecular Pathology. Genet. Med..

[39] Li M.M., Datto M., Duncavage E.J., Kulkarni S., Lindeman N.I., Roy S., Tsimberidou A.M., Vnencak-Jones C.L., Wolff D.J., Younes A. (2017). Standards and Guidelines for the Interpretation and Reporting of Sequence Variants in Cancer. J. Mol. Diagn..

[40] Schafer S., De Marvao A., Adami E., Fiedler L.R., Ng B., Khin E., Rackham O.J.L., Van Heesch S., Pua C.J., Kui M. (2017). Titin-Truncating Variants Affect Heart Function in Disease Cohorts and the General Population. Nat. Genet..

[41] Roberts A.M., Ware J.S., Herman D.S., Schafer S., Baksi J., Bick A.G., Buchan R.J., Walsh R., John S., Wilkinson S. (2015). Integrated Allelic, Transcriptional, and Phenomic Dissection of the Cardiac Effects of Titin Truncations in Health and Disease. Sci. Transl. Med..

[42] Hahnen E., Hauke J., Engel C., Neidhardt G., Rhiem K., Schmutzler R.K. (2017). Germline Mutations in Triple-Negative Breast Cancer. Breast Care.

[43] Ma D., Chen S.-Y., Ren J.-X., Pei Y.-C., Jiang C.-W., Zhao S., Xiao Y., Xu X.-E., Liu G.-Y., Hu X. (2021). Molecular Features and Functional Implications of Germline Variants in Triple-Negative Breast Cancer. JNCI J. Natl. Cancer Inst..

[44] Shi Y., Jin J., Ji W., Guan X. (2018). Therapeutic Landscape in Mutational Triple Negative Breast Cancer. Mol. Cancer.

[45] Wang Y.A., Jian J.-W., Hung C.-F., Peng H.-P., Yang C.-F., Cheng H.-C.S., Yang A.-S. (2018). Germline Breast Cancer Susceptibility Gene Mutations and Breast Cancer Outcomes. BMC Cancer.

[46] Hu C., Polley E.C., Yadav S., Lilyquist J., Shimelis H., Na J., Hart S.N., Goldgar D.E., Shah S., Pesaran T. (2020). The Contribution of Germline Predisposition Gene Mutations to Clinical Subtypes of Invasive Breast Cancer From a Clinical Genetic Testing Cohort. JNCI J. Natl. Cancer Inst..

[47] Lei C., Lu W., Li Y., Yang H., Zhang K., Wang N., Xuan L., Guo C. (2025). *SERPINA1* gene regulates the tumorigenesis and progression of breast cancer through PI3K/AKT signaling pathway and tumor immune microenvironment. Sci. Rep..

[48] Sukumar J., Kassem M., Agnese D., Pilarski R., Ramaswamy B., Sweet K., Sardesai S. (2021). Concurrent Germline *BRCA1*, *BRCA2*, and *CHEK2* Pathogenic Variants in Hereditary Breast Cancer: A Case Series. Breast Cancer Res. Treat..

[49] Pócza T., Papp J., Bozsik A., Grolmusz V.K., Nagy P., Patócs A., Butz H. (2025). Double Pathogenic or Likely Pathogenic Variants in Cancer Predisposition Genes in Hungarian Cancer Patients. Int. J. Mol. Sci..

[50] Kaprin A.D., Starinskiy V.V., Shakhzadova A.O., Zolotareva N.Y. (2025). Malignant Neoplasms in Russia in 2024 (Incidence).

