# Supplementary material for: Extended Germline Profiling of Cancer Predisposition and Homologous Recombination Repair Genes in 275 Russian Patients with Triple-Negative Breast Cancer
Source: Biomedicines. 2026 Aug 1;14(8):1742. doi: 10.3390/biomedicines14081742 (PMC13509342; doi:10.3390/biomedicines14081742)
Supplement: Supplementary file 1 [file biomedicines-14-01742-s001.zip › Supplementary figures.pdf]

Supplementary Figure S1. ***In silico*** **comparison of exome capture panel target coverage across MANE Select coding sequences.**

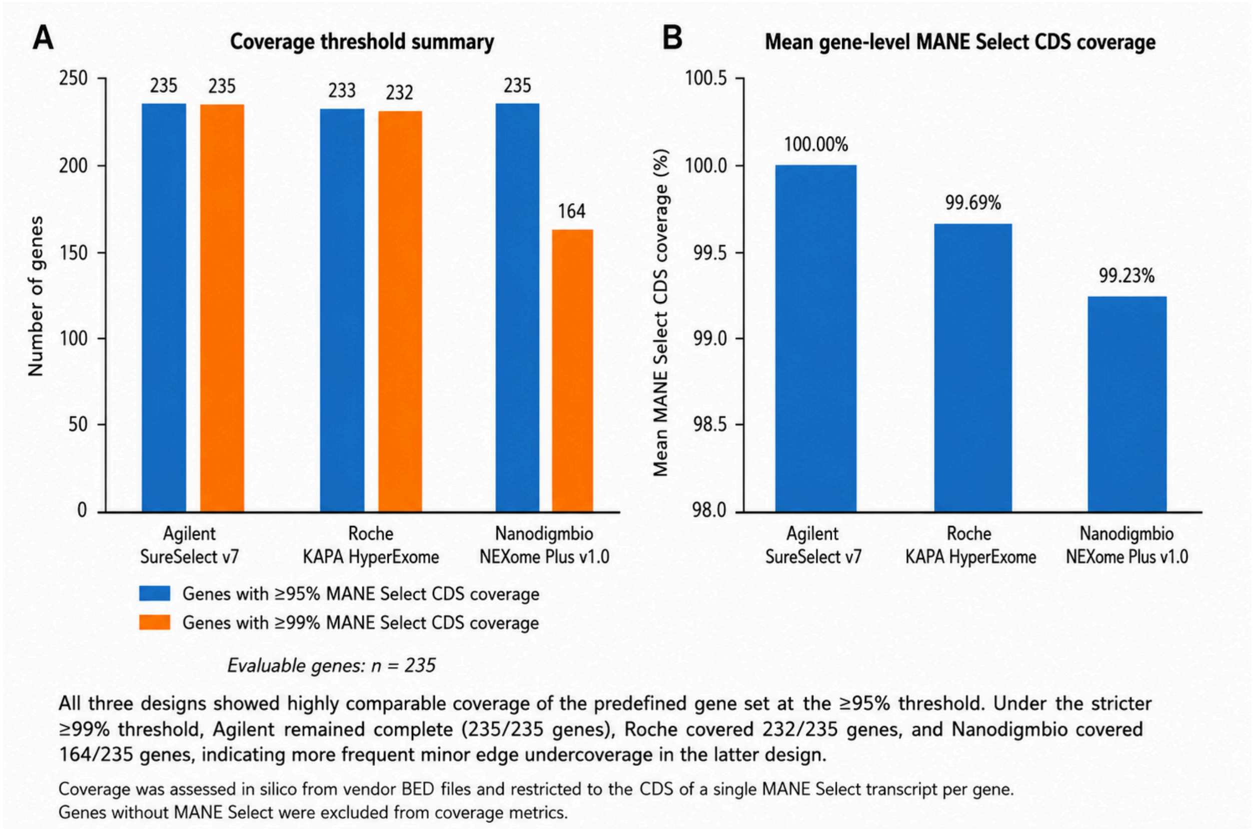

Supplementary Figure S2. VUS burden and age at diagnosis

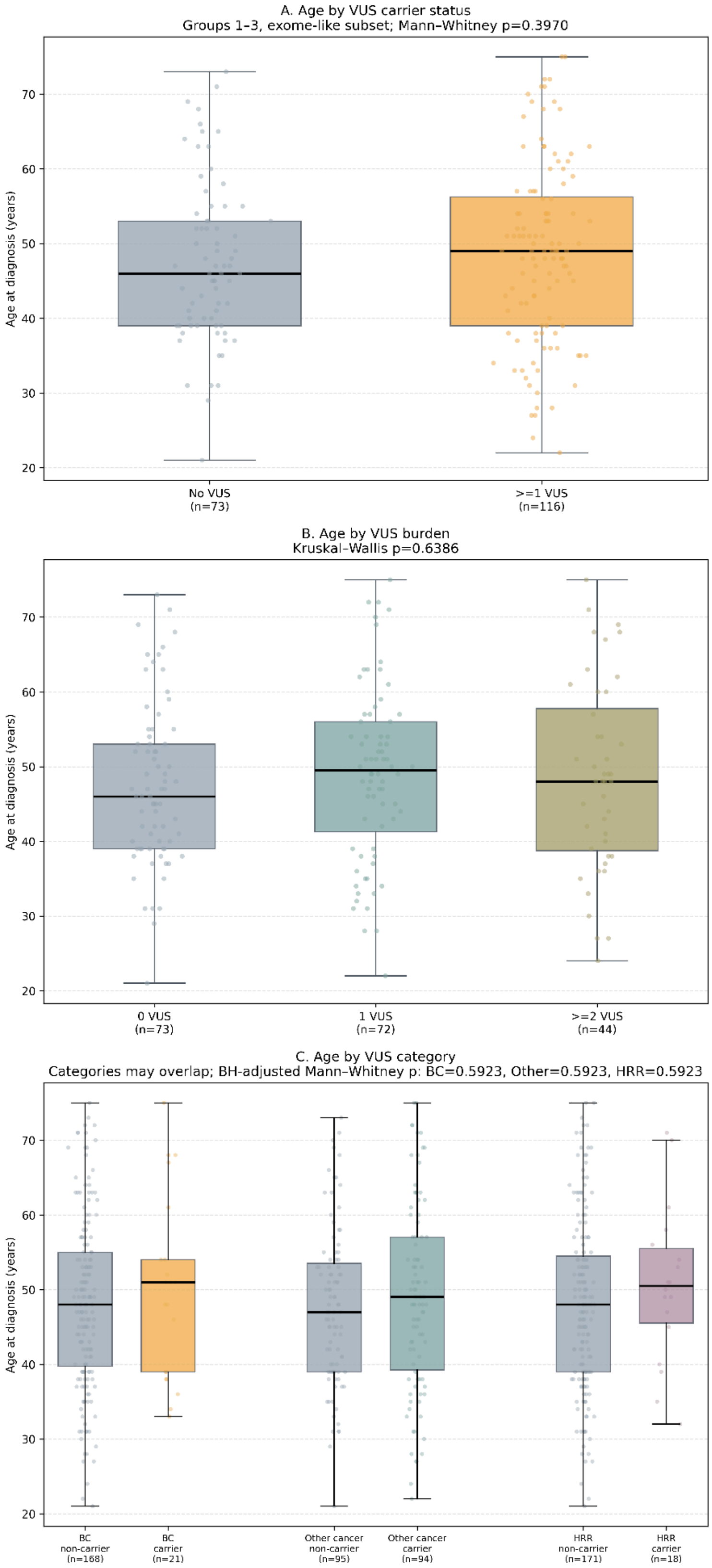

Supplementary Figure S1. In silico comparison of exome capture panel target coverage across MANE Select coding sequences. (A) Number of evaluable genes meeting ≥95% and ≥99% MANE Select CDS coverage thresholds for each exome capture design. (B) Mean gene-level MANE Select CDS coverage across the evaluated gene set. Coverage was assessed using vendor BED files and restricted to the CDS of one MANE Select transcript per gene. GSTT1 was excluded from coverage metrics because no corresponding MANE Select CDS interval was available in the annotation file used for this analysis.

Supplementary Figure S2. Association of VUS burden with age at TNBC diagnosis. The exploratory analysis was restricted to patients from the exome subset with available age data (n = 189). VUSes in Groups 1–3 were analyzed at the patient level. (A) Age distribution in patients with and without ≥1 VUS. (B) Age distribution according to VUS burden (0, 1, or ≥2 VUSes). (C) Age distributions by VUS category: breast cancer predisposition genes, other cancer-related genes, and additional HRR-related genes. Boxes indicate the interquartile range, horizontal lines indicate medians, whiskers extend to 1.5 times the interquartile range, and points represent individual patients. VUSes were not interpreted as pathogenic findings.
